# Real-Time Cuffless Continuous Blood Pressure Estimation Using 1D Squeeze U-Net Model: A Progress toward mHealth

**DOI:** 10.3390/bios12080655

**Published:** 2022-08-18

**Authors:** Tasbiraha Athaya, Sunwoong Choi

**Affiliations:** 1 Department of Computer Science, University of Central Florida, Orlando, FL 32816, USA; 2 School of Electrical Engineering, Kookmin University, Seoul 02707, Korea

**Keywords:** blood pressure (BP), Squeeze U-net, photoplethysmogram (PPG), mHealth, real time, deep learning

## Abstract

Measuring continuous blood pressure (BP) in real time by using a mobile health (mHealth) application would open a new door in the advancement of the healthcare system. This study aimed to propose a real-time method and system for measuring BP without using a cuff from a digital artery. An energy-efficient real-time smartphone-application-friendly one-dimensional (1D) Squeeze U-net model is proposed to estimate systolic and diastolic BP values, using only raw photoplethysmogram (PPG) signal. The proposed real-time cuffless BP prediction method was assessed for accuracy, reliability, and potential usefulness in the hypertensive assessment of 100 individuals in two publicly available datasets: Multiparameter Intelligent Monitoring in Intensive Care (MIMIC-I) and Medical Information Mart for Intensive Care (MIMIC-III) waveform database. The proposed model was used to build an android application to measure BP at home. This proposed deep-learning model performs best in terms of systolic BP, diastolic BP, and mean arterial pressure, with a mean absolute error of 4.42, 2.25, and 2.56 mmHg and standard deviation of 4.78, 2.98, and 3.21 mmHg, respectively. The results meet the grade A performance requirements of the British Hypertension Society and satisfy the AAMI error range. The result suggests that only using a short-time PPG signal is sufficient to obtain accurate BP measurements in real time. It is a novel approach for real-time cuffless BP estimation by implementing an mHealth application and can measure BP at home and assess hypertension.

## 1. Introduction

Fluctuations in blood pressure (BP) correlate strongly with vital organ damage in the case of high BP or hypertension [1]. Early detection and control of hypertension problems are crucial, but they rely on precise and accessible measurements [2]. According to statistics from the World Heart Federation (WHF), almost half of cardiac strokes are the reason for hypertension [3]. It also raises your chances of having a hemorrhagic stroke, cardiac failure, a heart attack, or chronic renal diseases [3,4]. By the time of 2025, the number of persons suffering from hypertension is intended to reach almost 1.5 billion. Any healthcare system would be overburdened by the consequences of this sickness [2]. Epidemiological studies reveal that hypertension prevalence and control have plateaued in developed countries since the middle of the 2000s [5,6]. As a result, proper blood pressure regulation is essential for preventing both primary and secondary coronary heart diseases; thus, introducing a reliable and comfortable method to measure BP values is mandatory.

The first and most reliable process of measuring continuous BP is the invasive or direct way, also called the invasive arterial BP (ABP). In order to monitor real-time continuous BP, a catheter is placed inside an artery for invasive ABP measurement. In this process, BP can be measured in every cardiac cycle, and variations in blood pressure can be tracked in a more precise way. Hence, it is widely regarded as the gold standard in the field of BP measurement methods [7]. However, this method requires arterial puncture. The kits used are non-reusable and have a high risk of complications and infections. Moreover, the measurement needs to be performed by trained professionals; thus, this method is only used for critically ill patients. The difficulties associated with the invasive methods initiate the necessity of introducing noninvasive measurement methods.

Traditionally and most commonly, noninvasive cuff-based auscultatory and oscillometric measurements are used to check BP. The measured results are expressed as systolic BP (SBP) and diastolic BP (DBP). To measure BP, cuff-based equipment is widely used in houses and hospitals [8]. Even a wireless cuff-based BP measurement device with a smartphone was also proposed [9]. However, the measurement process is discontinuous and takes a long time, and the result varies for different cuff sizes. Furthermore, continuous BP measurement is impossible because repetitive cuff inflation causes discomfort. With technological advancement, research has been conducted widely to measure BP more accurately at a low cost. Almost everyone has a smartphone in their hand. Wearable devices are becoming increasingly popular. So, signals acquired using different sensors for accurate BP measurement are nowadays a wide research topic. It also paves the way for real-time continuous BP measurements.

Recently, an increase in interest has been observed in the prediction of cuffless real-time BP values to enable an easy and comfortable way of predicting BP in real time by using features of different easily measurable biosignals. Several noninvasive cuffless BP estimation methods have been proposed using photoplethysmogram (PPG) and electrocardiogram (ECG) signals, including ultrasound-based [10], pulse transit time (PTT)-based [11,12,13,14,15], and feature-based approaches [8,16,17,18,19].

The ultrasound-based technique was developed for continuous real-time BP measurement; however, higher complexity existed [19]. Reviewing the complexities, a comparably lower complex PTT-based approach [11,12,13,14,15] was studied extensively for a long time. PTT is the time needed for the pulse pressure waveform of the artery to reach a peripheral site (e.g., fingertip) from the semilunar valves. Usually, it is measured from the start of the R-wave on the ECG signal to the arrival of the PPG signal at the fingertip [20]. Thus, the time can be calculated by using two channels of biosignals, and frequent calibrations are needed to ensure prediction accuracy [12].

Furthermore, feature-based approaches [8,16,17,18,19,21] have been proposed to improve the PTT-based BP estimation methods. In a feature-based approach, multiple features that influence BP are extracted from the ECG and PPG signals and merged to obtain BP values with improved accuracy. Different machine-learning models, such as the random forest, support vector machine, and neural networks, are used to predict BP values by using the extracted features. However, two channels of biosignals are required for most of these methods. Feature-based models using one-channel ECG [18] or PPG [22] signals exhibit an inferior performance. Moreover, additional sensors are needed to acquire ECG signals that are impossible to acquire by using smartphones. Feature-based methods need complicated feature extraction and selection processes that urge high-quality waveforms to extract proper features [23]. Moreover, it is difficult to get a generalized model by using a definite number of features. A study also used different BP and bodily parameters to predict BP values by using different machine learning models and ensemble methods [24]. However, they obtained very low accuracy.

Deep learning (DL) approaches can automatically learn useful features. Based on this property of DL, S. Baker et al. proposed continuous BP estimation by using raw ECG and PPG signals [25], and K. Qin et al., G. Slapničar et al., and T. Athaya et al. proposed that estimation techniques using only raw PPG signals can be actualized [23,26,27]. Using a one-channel raw PPG signal is more suitable than using two channels of biosignals to estimate continuous BP by using smartphones and wearable devices [28]. G. Slapničar et al. proposed a complex GRU-based spectro-temporal deep neural network by using both derivatives and information of PPG signal frequency domain information with lower accuracy [26]. K. Qin et al. and T. Athaya et al. predicted BP waveform by using raw PPG signal, and from the predicted BP waveform, SBP, DBP, and ABP values were predicted [23,27]. However, T. Athaya et al. used the U-net model, which needs many parameters and is thus unsuitable for real-time mHealth applications [27]. Furthermore, K. Qin et al. used regularized deep autoencoder (RDAE), which provides lower accuracy [23]. Moreover, the model is not generalized for every BP value, and calibration is needed to obtain a better performance. Although continuous methods are proposed in these studies, none of the methods ensures real-time BP estimation.

Unlike the abovementioned methods, herein, the prediction of real-time continuous BP values and implementation using a smartphone application are proposed. A real-time application-friendly Squeeze U-net model was used to predict the BP values by directly feeding a one-channel raw PPG signal as the model input. The proposed model ensures a user-friendly and generalized system to measure BP values ubiquitously and unobtrusively without calibration to obtain accurate results. The proposed architecture of the model provides low computational complexity and higher efficiency to estimate BP. The main contributions herein are as follows:

- Real-time BP values are estimated by using a single-channel raw PPG signal as the input of the mobile application-friendly modified 1D Squeeze U-net model.
- This study is a novel approach to implementing a DL 1D Squeeze U-net model in an mHealth application, using PPG signals without any feature selection to generate BP values with high accuracy and at no cost.

The rest of the study is organized as follows: Section 2 describes the used materials and proposed methodology. Section 3 shows the results from using the chosen materials and methods. Section 4 discusses the implications of the proposed approach. Finally, Section 5 concludes the study.

## 2. Materials and Methods

### 2.1. Data Acquisition

**Figure 1:**
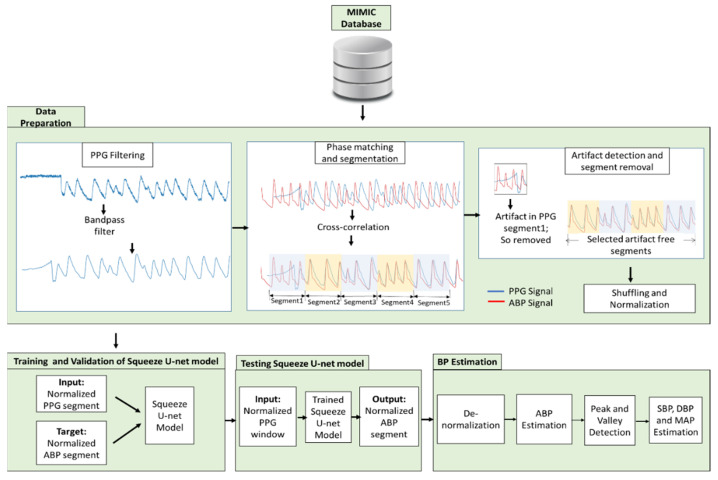
Proposed methodology to train, validate, and test the proposed Squeeze U-net model.

DL works best when huge amounts of data are utilized to train, validate, and test the models. The invasive ABP and fingertip PPG signals were needed herein. The first step of Figure 1 is data collection. The signals are acquired from two publicly available popular databases containing health-related data. The databases contain simultaneous recordings of the fingertip PPG, ABP, one or more ECG, pulmonary arterial pressure, and central venous pressure signals. The databases are as follows:

- Multiparameter Intelligent Monitoring in Intensive Care (MIMIC) database [29].
- Medical Information Mart for Intensive Care III (MIMIC-III) waveform database [30,31].

Herein, 45 individuals of the MIMIC-I dataset and a subset of 55 individuals of the MIMIC-III waveform dataset comprising fingertip PPG and ABP signals with a 125 Hz sampling rate were used. Finally, recordings of 100 individuals were acquired for the whole experiment.

### 2.2. Data Preparation

The next step in Figure 1 is data preparation. The Squeeze U-net was used for predicting BP values. As the Squeeze U-net is the SqueezeNet type of the U-net model, the data preparation for training and validation of the model is the same as the process used for the U-net model [27]. First, the PPG signal acquired from the two databases is filtered by using an Equiripple FIR bandpass filter using a cutoff frequency of 0.5–8 Hz. Frequencies of less than 0.5 Hz and higher than 8 Hz are considered to be noise and are, thus, filtered out.

In the databases, the ABP signals are obtained from the brachial artery, and the PPG signals are obtained from the digital artery. As blood enters first in the brachial artery and then in the digital artery, a phase difference is shown between the two signals, even if they are recorded simultaneously. To remove the delay between the two signals measured from different sources, the filtered PPG signals are phase matched with the respective ABP signals, using the cross-correlation technique for properly replicating the output ABP signals, using PPG signals as the input. Afterward, the phase-matched signals are divided into segments of 256 samples.

Next, the segments are checked for artifacts and divided into two classes: acceptable and unacceptable signals. The unacceptable segments with artifacts are removed from the final dataset by using a random forest machine-learning model [32]. In Reference [32], real-time artifact detection of PPG signals is shown. As our system is also in real time, we used the approach. As much variation of the PPG signal as possible was maintained in the final dataset to enable the generalization of the trained model.

Next, the data were shuffled for unbiased training of the model. Finally, the acquired signals were normalized by using Equation (1) to nullify the variation of PPG and ABP signal values for different individuals [27,33].

(1) $$x_{norm\left(i\right)}=\frac{\left(x_{i}-x_{min}\right)}{x_{max}-x_{min}},$$

where *x_i_* refers to the *i*th signal segment, and $x_{max}$ and $x_{min}$ are the highest and lowest values of all segmented signals, respectively. Note that $x_{max}=2.9\;\mathrm{mV}$ and $x_{min}=-2.4\;\mathrm{mV}$ for PPG and $x_{max}=192.24\;\mathrm{mmHg}$ and $x_{min}=50.33\;\mathrm{mmHg}$ for ABP.

### 2.3. Proposed Architecture of Squeeze U-Net Model

The second step of Figure 1 is the training and validation of the proposed Squeeze U-net model. The proposed model is modified to one-dimensional (1D) convolutional blocks despite comprising 2D convolutional blocks such as the original one. Inspired by the SqueezeNet type of the U-net model for biomedical image segmentation, the proposed model has been presented to ease the development of deep neural networks on embedded systems, while minimizing the computational and memory requirements, especially for real-time smartphone applications [34].

The SqueezeNet fire module [35] architecture was introduced in both the contracting and expansion paths while designing the proposed model. Note that the convolution blocks of the fire modules were changed to 1D for our proposed modified model. The initial depth-wise convolution of the fire module reduces the number of channels, and this is compensated by two parallel convolutions in the expansion units, with each convolution having half the number of output channels of that of the squeeze unit. Both parallel convolutions support avoiding feature loss and vanishing gradients, which can occur when the number of channels is reduced [36].

**Figure 2:**
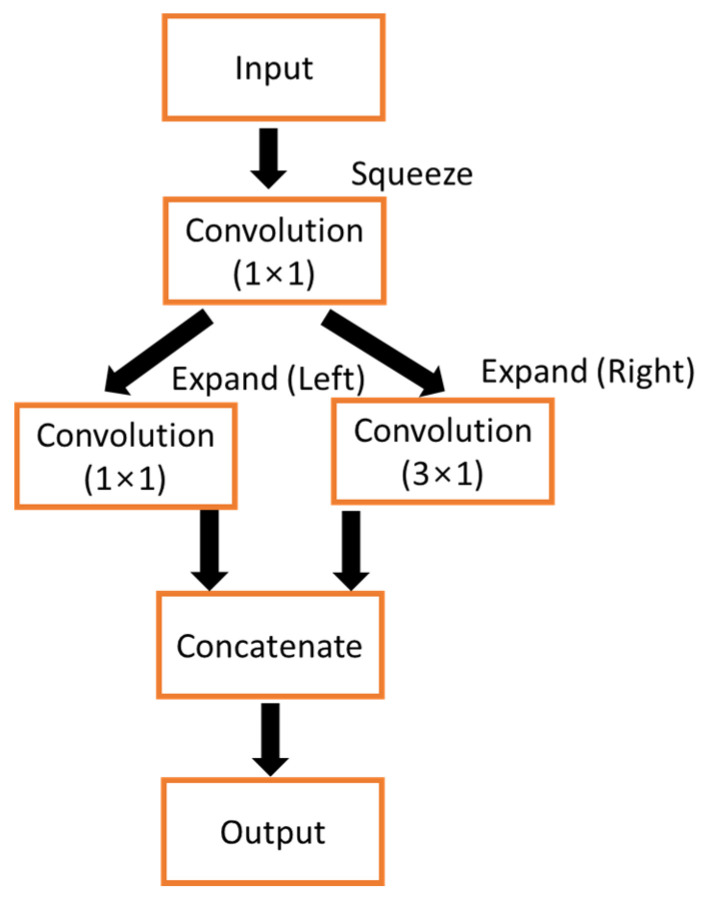
Structure of the proposed fire module of Squeeze U-net.

The fire module (Figure 2) comprises a squeeze, two expansion convolution units, and a concatenate unit. The squeeze unit has a 1 × 1 convolution filter. The output of this unit expands the convolution units of 1 × 1 filters (left) and 3 × 1 filters (right) in parallel. The convolution filters in the 1 × 1 squeeze unit, 1 × 1 expansion unit, and 3 × 1 expansion unit are denoted as s_1×1_, e_1×1_, and e_3×1_, respectively. While using fire modules, s_1×1_ is set less than (e_1×1_ + e_3×1_) to limit the number of input samples. In CNN, to decrease the number of parameters, it is necessary to minimize the number of input samples. Thus, the output of the expansion units goes to the concatenate unit to give (e_1×1_ + e_3×1_) filters. The result of the concatenate unit is the output of the fire module.

**Figure 3:**
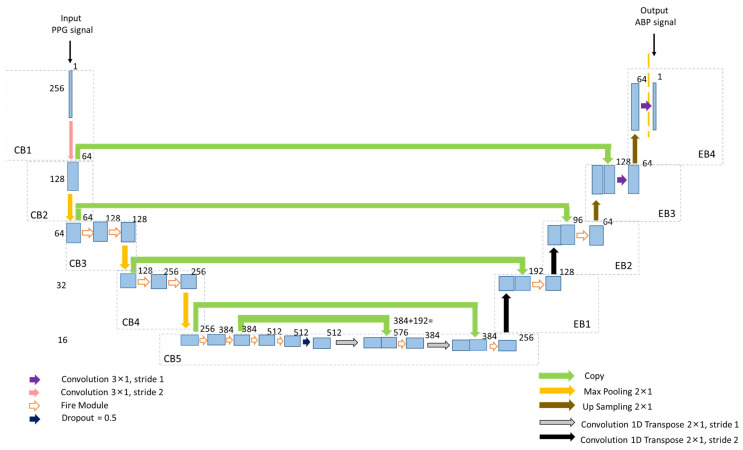
The proposed architecture of one-dimensional (1D) Squeeze U-net DL model.

The proposed 1D Squeeze U-net model is illustrated in Figure 3. The model has contracting and expansion paths on the left and right sides, respectively. The contracting and expansion paths comprise several contraction blocks (CBs) and expansion blocks (EBs), as the U-net does. The numeral on the left side of the blocks illustrates the size of the input vector or sample. The numeral on the right side of every output unit illustrates the size of the feature vector.

#### 2.3.1. Contracting Path

In the first contraction block (CB1) of the proposed model, the PPG segment of 256 samples was used as the input. The input is passed to the 3 × 1 convolution unit with stride 2 in CB1. Striding helps decrease the number of samples to 128 and increases the model’s expressiveness. The feature vectors are 64 after the 3 × 1 convolution operation with stride 2 in CB1. In CB2, the output of the convolution unit of CB1 goes to the 2 × 1 max-pooling layer. No convolution operation is performed in CB2. Max-pooling halves the samples from 128 to 64, and the number of feature vectors remains the same. The CB2 output is passed into two fire modules and one 2 × 1 max-pooling in CB3.

In the Squeeze U-net network, fire modules are introduced for down-sampling units in the contracting path. The fire modules are used to reduce the total parameter number for the model. After passing the first fire module, the number of feature vectors doubles and remains the same after the next fire module and max-pooling operation. The fire modules of CB3 have 16 squeeze filters (s_1×1_) and 64 expand filters (e_1×1_ and e_3×1_). Then the 4th block (CB4) of the contracting path comes, which has a similar structure as CB3. The fire modules of CB4 have 32 s_1×1_, 128 e_1×1_, and e_3×1_, making the number of feature vectors 256 after two consecutive fire modules. The last block (CB5) of the contracting path is max-pooled and passes four fire modules. The first two fire modules have 48 s_1×1_, 192 e_1×1_, and e_3×1_. The other two fire modules have 64 s_1×1_, 256 e_1×1_, and e_3×1_. Next, the dropout is performed, and the output is passed into a 1D convolution transpose unit with stride 1. The output of this block is concatenated with the output of the second fire module of block CB5. The concatenated output goes to a fire module. This fire module has 48 s_1×1_, 192 e_1×1_, and e_3×1_. The output of this fire module again passes into the 2 × 1 1D convolution transpose unit with stride 1. The output of this block is concatenated with the input of the first fire module of CB5, and the concatenated result is passed into a fire module. This fire module has 32 s_1×1_, 128 e_1×1_, and e_3×1_. At the end of CB5, the number of feature vectors becomes 256. Similar to the structure of U-net, this Squeeze U-net network uses the Leaky ReLU activation function in every convolution unit.

#### 2.3.2. Expansion Path

The expansion path has four expansion blocks, which are denoted as EB1, EB2, EB3, and EB4. Each EB1 and EB2 block comprises a 2 × 1 1D convolution transpose unit with stride 2. The output of the convolution transpose unit is passed into the concatenation units. After passing the concatenation units, in EB1 and EB2, the number of feature vectors becomes 192 and 96, respectively. The EB1 and EB2 blocks of the expansion path also have fire modules. The outputs of the concatenation units go to these fire modules. The EB1 fire module of the expansion path has 16 s_1×1_, 64 e_1×1_, and e_3×1_, and EB2 has 16 s_1×1_, 32 e_1×1_, and e_3×1_. After passing the fire modules in EB1 and EB2, the number of feature vectors becomes 128 and 64, respectively. Each EB3 and EB4 block has a 2 × 1 up-sampling unit. The output of the up-sampling unit of EB3 is concatenated with the input layer of CB2. The output of the concatenated unit passes into a 3 × 1 convolution unit of stride 1 in EB3. The EB3 output goes through a 2 × 1 up-sampling unit in EB4. The output of the EB4 up-sampling unit again passes to the 3 × 1 convolution unit in EB4 and gives the output ABP window of 256 samples.

### 2.4. Training and Testing of Squeeze U-Net Model

The normalized PPG signals are used as the input of the 1D Squeeze U-net model to obtain the normalized ABP-like signals in the testing phase (Figure 1). Of the overall pre-processed data, 70% were used to train the network, 15% to validate, and the left 15% to test. The training, validating, and testing data were totally different from each other. The network is trained and tested by using Keras. The training data are needed for iterative model training until the maximum number of epochs is reached to obtain a generalized model with the highest performance. The network performance is monitored dynamically. The validation is performed to fine-tune the hyperparameters and stop the training process when no improvement is seen to generalize the network. Then the testing was performed by using the highest-performing network structure with the test dataset. 

The Adam optimizer was used to train the network, and the chosen loss function was mean squared error. For the Squeeze U-net model, the learning rate was set at 10^−4^, and the batch size was set to 10. The learning rate and batch size of the network were both determined through experimentation. When there was no improvement in the consecutive six epochs, early stopping was applied, and the Squeeze U-net model’s training was ended after 33 epochs. The epoch performance in reduced validation loss was automatically saved for each epoch in this training method. An NVIDIA GTX 1080 Ti 10 GB graphics card with a GPU server and 257 GB RAM was used in this experiment. Python language was used to write all the codes.

### 2.5. BP Estimation

In the final step, the BP values are estimated as depicted in Figure 1. The obtained signal was denormalized by using the stored denormalization factors of the training phase. Next, a standard peak detection algorithm [37] was used to estimate the SBP and DBP values. From these values, the mean arterial pressure (MAP) was estimated by using Equation (2) [38].

(2) $$MAP=\frac{1}{3}SBP+\frac{2}{3}DBP$$

## 3. Results

### 3.1. Performance of Squeeze U-Net Model

The dataset for performance evaluation is distributed from different BP values of all ranges. The data summary is displayed in Table 1. We tried making a dataset comprising all kinds of BP values for proper result evaluation.

Different performance matrices have been used in different studies to show the performance of the model. We tried incorporating all performance matrices to evaluate the performance of the proposed model for real-time BP estimation. The mean error (ME), mean absolute error (MAE), standard deviation (STD), root mean square error (RMSE), and Pearson’s correlation coefficient (r) evaluation factors were used [39].

**Figure 4:**
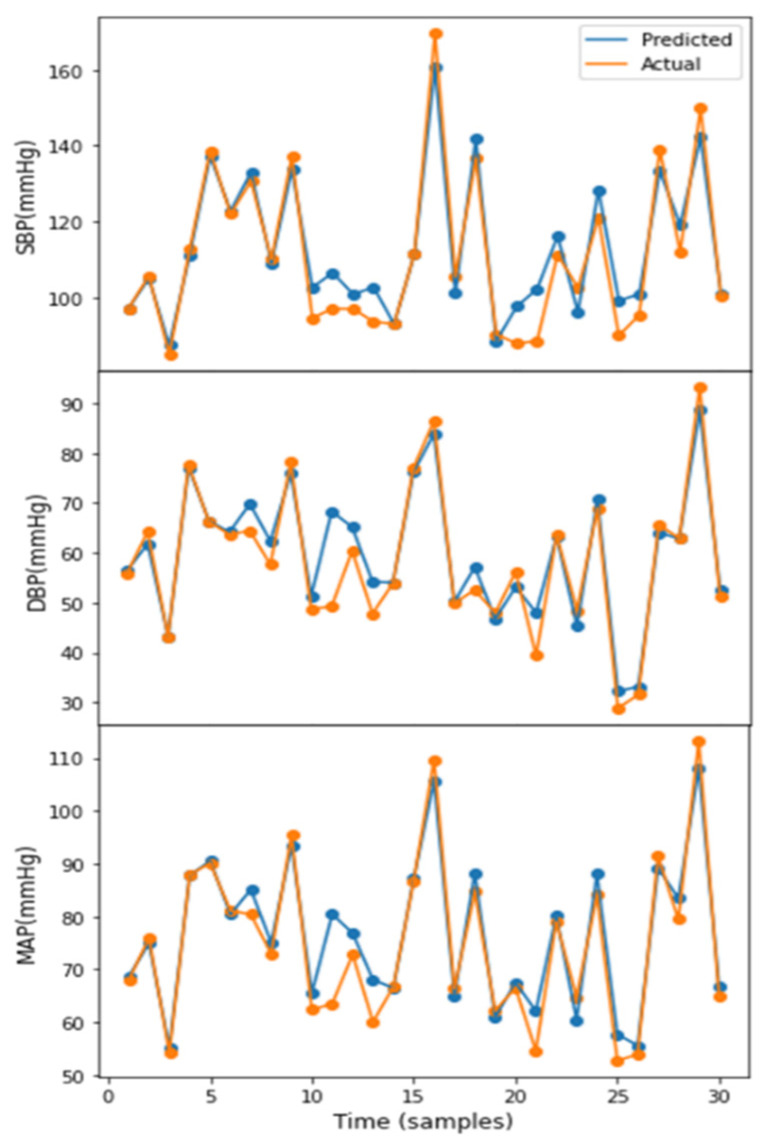
An example of real-time systolic blood pressure (SBP), diastolic blood pressure (DBP), and mean arterial pressure (MAP) estimation.

The performance details of the proposed Squeeze U-net model to estimate the real-time SBP, DBP, and MAP values are presented in Table 2. In all three cases, the evaluation factors provide good values, indicating that the network can be used to estimate SBP, DBP, and MAP accurately. The real-time SBP, DBP, and MAP estimation results using the Squeeze U-net model are illustrated in Figure 4. The figure shows the similarity between the predicted and actual values.

**Figure 5:**
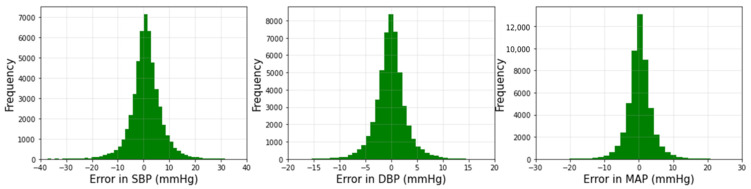
Histograms of error for estimated real-time SBP, DBP, and MAP values.

Figure 5 shows the histogram plots of the prediction error, using the 1D Squeeze U-net model for SBP, DBP, and MAP values. For three cases, the error values lie at zero, indicating an accurate prediction of most values. The prediction accuracy of MAP is the highest.

**Figure 6:**
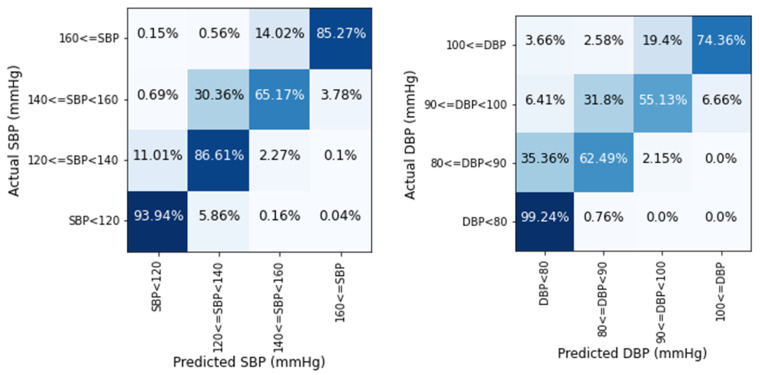
Prediction accuracy of SBP and DBP values in four BP stages.

Figure 6 shows that the proposed network performs well in all four BP stages: normal, prehypertension, hypertension stage-1, and hypertension stage-2 [40]. In the normal stage, the SBP and DBP estimation accuracy rates are 93.94% and 99.24%, with the latter being the highest. The SBP prediction accuracy is better in the prehypertension stage than that of DBP. For hypertension stage-1, the percentage of prediction accuracy is low for both SBP and DBP. For SBP 30.36% and DBP 31.8%, the values are inaccurately predicted as prehypertension stage. Despite minor variations in predicting hypertension stage-1, the prediction accuracy for hypertension stage-2 is fairly good. For a vast amount of data, relatively fewer values are discovered in a deviation area of more than 20 mmHg, which is trivial.

**Figure 7:**
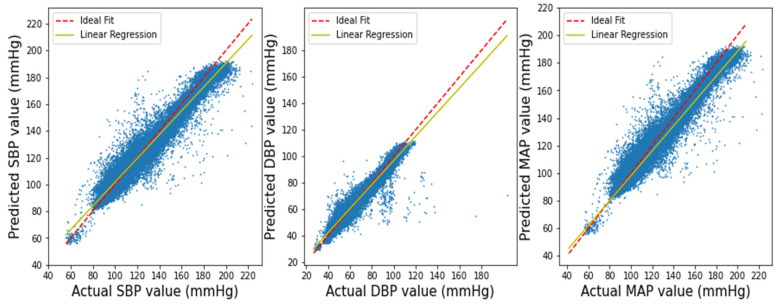
Linear regression plot for SBP, DBP, and MAP results.

Figure 7 depicts the scatter plots of our anticipated outcome vs. the actual SBP, DBP, and MAP values. It has been demonstrated that the acquired result yields a linear relation, demonstrating that the anticipated outcome is largely correct, except in some situations.

**Figure 8:**
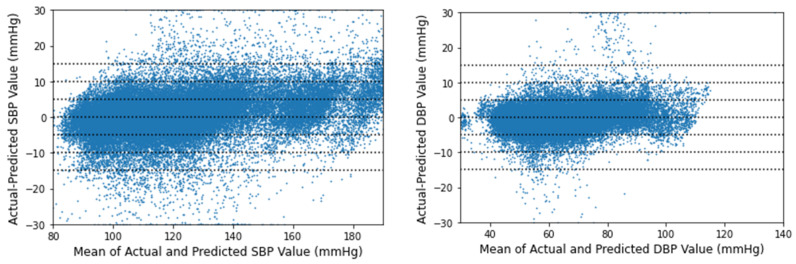
Bland–Altman analysis for real-time SBP and DBP values.

The Bland–Altman analysis for the proposed deep learning model is displayed in Figure 8 according to the AAMI norm [41]. No Bland–Altman plot exists for MAP in the AAMI norm. The *x*-axis shows SBP pressures ranging from 80 to 190 mmHg and DBP pressures ranging from 30 to 140 mmHg. The *y*-axis represents inaccuracies ranging from 30 to +30 mmHg. From 15 to +15 mmHg, reference horizontal dotted lines are provided at 5 mmHg intervals. The mean of each actual BP and its related projected BP is represented by a point across their difference. Differences more than 30 mmHg are shown at 30 mmHg, and differences less than 30 mmHg are plotted at 30 mmHg. Most SBP and DBP discrepancies are between ±5 mmHg in both scenarios.

### 3.2. Result Evaluation and Comparison

Although several studies are found in the web of science that can estimate BP or continuous BP, few studies predicted BP values in real time. The results were compared with recent works that predicted continuous BP or real-time BP. Furthermore, the comparison of the obtained results was actualized by using two established standards: AAMI [42] and British Hypertension Society (BHS) standards [43].

To satisfy the AAMI error range, the testing device used to measure BP values must have ME ≤5 mmHg and STD ≤8 mmHg, with above 85 subjects. Table 3 compares our results based on the AAMI standard. In References [25,44], it is observed that calculating the ME gives incorrect estimations, as a lower ME may result in a higher MAE. Herein, both ME and MAE were included in all papers. The standard norm for AAMI is passed for all three BP values using our 1D Squeeze U-net model. Our results obtained a comparatively better MAE and STD than all other papers.

The testing device is divided into three grades (A, B, or C) based on the conditions of the BHS grading standard [43] (Table 4). The criterion of a device to satisfy the BHS standard error range is that it must reach the minimum Grade B in estimating the SBP and DBP values. According to the BHS grading standard criteria, the proposed model obtained grade A in predicting SBP and DBP values. The SBP, DBP, and MAP results are shown in Table 4 according to the BHS standard. References [17,18,19] obtained grade B in predicting the SBP value. Reference [23] obtained grade C for SBP prediction without calibration. Since a generalized model is desired, calibration-free results are considered. Only References [25,27] obtained grade A for SBP prediction. All papers obtained grade A in predicting the DBP values, indicating that DBP correlates strongly with the PPG and ECG signals. Compared to the other models, our work obtained grade A for all three values, indicating the superiority of our results over those in References [17,18,19,23], according to the BHS standard.

Comparisons based on real-time BP measurement, methodology, dataset, RMSE, and Pearson’s correlation coefficient (r) with recent papers are presented in Table 5. Y.-H. Li et al. [17] proposed the BiLSTM network that uses ECG and PPG features to predict SBP and DBP values in real time. They showed an implementation of their real-time algorithm in the MATLAB interface. ResNet with a bidirectional LSTM was used by F. Miao et al. [18]. The 50-layered networks are combined to extract features from preprocessed signals. The BP values were estimated by using the features. The study showed no device implementation of their real-time algorithm. In contrast, F. Miao et al. used a multi-sensor regression algorithm with their self-made database, using both ECG and PPG signals from multiple sensors [19]. They used a USB port to send the digital signal to show it on a personal computer in real time. Unlike the feature-based works, S. Baker et al. combined the CNN and LSTM models with raw PPG and ECG signals to predict continuous BP [25]. However, their method is not in real time, and the correlation coefficient is comparatively low. The abovementioned studies either used a combination of two signals or ECG signals. However, as mentioned before, the ECG signal is complicated and cost ineffective. The signal cannot be obtained by using smartphones or smartwatches. To acquire the ECG signal, additional sensors are needed.

Unlike in these works, K. Qin et al. and T. Athaya et al. used raw PPG signals, using regularized convolution-based deep autoencoder (RDAE) and U-net models, respectively, to obtain continuous BP values [23,27]. The result of Reference [23] was unsatisfactory and needs calibration for improvement. However, the performance of Reference [27] was good, but it needs several parameters, operations, and prediction time (Table 6) to make it unsuitable for real-time implementation. Both works cannot measure BP in real time.

Different from the stated methods, our 1D Squeeze U-net method can predict BP values in real time by using raw PPG signals, with comparatively better accuracy and lower computational complexity; it is also suitable for mHealth application implementation. Table 6 showed the number of parameters, model size, number of operations, and prediction time of the proposed model which is comparatively lower to get better accuracy. As we used the proposed system to implement in an android device, keeping the computational complexity lower is very important.

### 3.3. mHealth Application

The computationally efficient Squeeze U-net model was used to calculate the real-time continuous BP, using an android application. Note that no other related works used an android application to predict real-time BP values, as per our knowledge. All stages of prior signal processing are performed in the android studio platform. The trained Squeeze U-net model is loaded by using TensorFlow Lite [45]. This model is used to predict SBP, DBP, and MAP values for new subjects.

**Figure 9:**
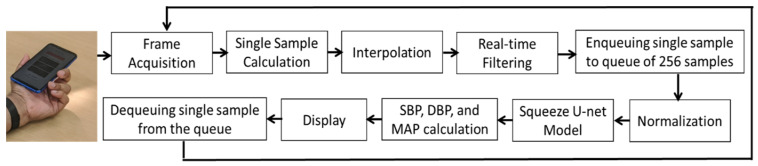
Implementation of real-time PPG signal filtering and BP values estimation.

For the implementation phase, the filtering process differs from the training phase. The PPG data are recorded by using the back camera of an android smartphone. A real-time Equiripple FIR bandpass filter is used in the implementation phase to estimate the BP values in real time. The real-time implementation process is shown in Figure 9. Placing the fingertip in the back camera, frames are acquired from the camera. Each time, a signal sample is calculated from the frame by taking the average of the red channel. The sampling frequency of the recorded signal using an android back camera is 30 Hz. The sampling frequency of the recorded signals must match the trained model; thus, linear interpolation [46] is performed with the recorded PPG signals [32]. Real-time Equiripple FIR bandpass filtering is performed on the red channel of the PPG signal by using the same filter coefficients used in the training phase. A single sample of the filtered PPG signal is taken in a 256 fixed-size queue. The segment is then normalized and used as the input of the trained Squeeze U-net model to get the predicted signal of 256 samples. Then the denormalization, peak detection, and estimation of BP values are performed sequentially.

**Figure 10:**
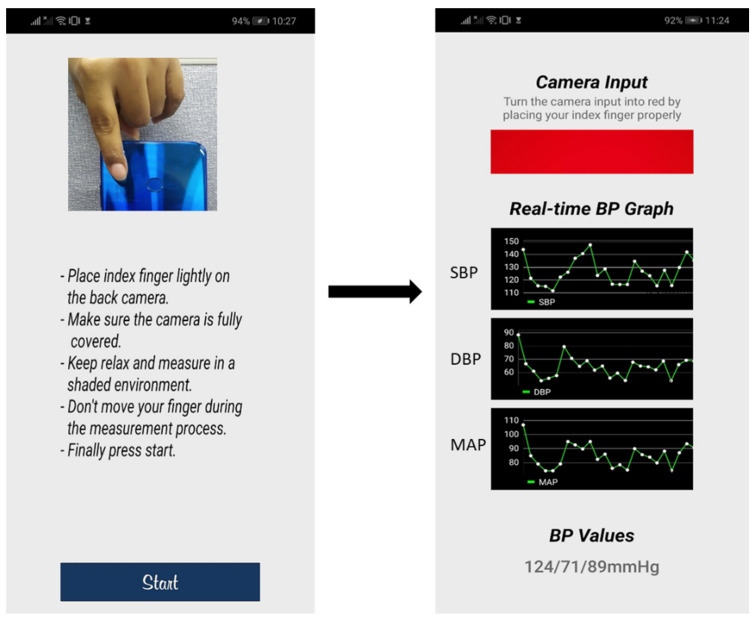
Real-time BP measurement using mHealth application.

The step-by-step real-time BP measurement process using this smartphone application is shown in Figure 10. By placing the index finger lightly on the camera, the PPG signal is acquired by using the rear camera of the smartphone to see the BP values in real time. To obtain accurate measurement results, neither the finger nor camera should not be moved until the measurement is completed. The graphs and BP values (Figure 10) are updated every second.

## 4. Discussion

The analysis of the results evidently provides comparable performances for real-time SBP, DBP, and MAP predictions. Mobile health diagnosis has been proven to be successful and scalable in identifying and managing chronic diseases. By utilizing the optical and computational power of smartphones, physiological information may be assessed from the shape of pulse waves, and, hence, it can estimate BP without using a cuff. So, the proposed Squeeze U-net model that uses a smartphone application can easily measure real-time BP values without any cost and replace cuff-based devices. However, individual variations present unique challenges to the robustness and generalized prediction ability of the prediction model. Thus, the application needs to be tested with the data of persons of different ages, BP levels, and genders, and then it can be commercially used.

Note that PPG signals can have different kinds of artifacts, and the signal quality varies from person to person based on the muscle layer’s volume and thickness, dermal and epidermal layers of skin, and volume of subcutaneous fats in a cross-section of the skin. The estimation result varies for the difference in finger pressure because the PPG signal is sensitive to finger pressure [47]. These parameters affect the estimation result. Although our study tried accumulating all kinds of PPG signals for a generalized model, some uncertainties still remain that might result in a high error level. Thus, the preliminary target of this application is for home use. However, if the proposed model is calibrated for each person, it should provide higher accuracy in real-time BP prediction, as the individual calibration process includes uncertain parameters.

In this study, the data from ICU patients were used. Because of different diseases, ICU patients exhibit abnormal PPG signals; thus, more normal subjects need to be included in the study in order to obtain more reliable predictions. Furthermore, the model’s response to the sudden change in BP values has not yet been explored, which is a challenging topic that can be explored in further studies. In the future, the model can be trained with an equal amount of data from patients in the four BP stages shown in Figure 6. Individual calibration can be added to remove the uncertainty of individual parameters and to control the sudden change in BP values. We will also perform research to set up a system so that the finger movement can be controlled. This can help the system to be used not only at home but also in hospitals and medical settings for regular and accurate prediction.

## 5. Conclusions

Herein, a novel 1D Squeeze U-net model that is efficient for android smartphones was proposed to predict real-time continuous BP, using a raw PPG signal, with simple preprocessing. The performance of the DL model was tested on 100 individuals of MIMIC and MIMIC-III waveform databases and provided promising results in real-time BP estimation. When checking the compliance of our model with the standards defined by the healthcare organizations AAMI and BHS, our network achieved impressive performance. The computationally efficient model was implemented in an android application that can measure BP in real time. After performing certain tests, we have determined that the application can be a useful tool for detecting hypertension in various circumstances, such as low-income nations, where smartphones are widely available and access to healthcare is limited, as they can have access to this application without any cost. Overall, our results show convincing evidence that the proposed Squeeze U-net model that uses a raw PPG signal can be reliably used for real-time BP estimation, thus incorporating real-time continuous BP value prediction in mHealth applications.

## Figures and Tables

**Table 1:** Summary of data used for testing network performance.

BP Values	Min (mmHg)	Max (mmHg)	Mean (mmHg)	STD (mmHg)
SBP	75.69	192.24	130.05	23.79
DBP	50.33	111.23	64.66	13.28
MAP	62.69	130.74	84.08	15.61

**Table 2:** Performance detail of Squeeze U-net models for systolic blood pressure (SBP), diastolic blood pressure (DBP), and mean arterial pressure (MAP) values measurement.

BP Values	ME (mmHg)	MAE (mmHg)	STD (mmHg)	RMSE (mmHg)	r
SBP	−1.002	4.42	4.78	6.50	0.970
DBP	0.019	2.25	2.98	3.73	0.964
MAP	−0.315	2.56	3.21	4.10	0.971

**Table 3:** Comparison with related works based on AAMI standard.

Works	BP Values	MAE	ME	STD	Result
AAMI [42]	BP		≤5	≤8	Passed
[17]	SBP	6.726	4.638	14.505	Failed
	DBP	2.516	3.155	6.442	Passed
	MAP	-	-	-	-
[18]	SBP	7.10	−0.11	9.99	Failed
	DBP	4.61	−0.03	6.36	Passed
	MAP	4.66	−0.01	6.29	Passed
[19]	SBP	6.13	1.62	7.76	Failed
	DBP	4.54	1.49	5.52	Passed
	MAP	4.81	1.53	6.03	Passed
[25]	SBP	4.41	-	6.11	Failed
	DBP	2.91	-	4.23	-
	MAP	2.77	-	3.88	-
[23]	SBP	7.945	1.447	10.375	Failed
	DBP	4.114	−0.417	5.504	Passed
	MAP	3.834	0.204	5.130	Passed
[27]	SBP	3.68	-	4.42	-
	DBP	1.97	-	2.92	-
	MAP	2.17	-	3.06	-
This work	SBP	4.42	−1.002	4.78	Passed
	DBP	2.25	0.019	2.98	Passed
	MAP	2.56	−0.315	3.21	Passed

**Table 4:** Comparison with related works based on British Hypertension Society (BHS) protocol.

Works	BP Values	Cumulative Error (%)			Grade
		≤5 mmHg	≤10 mmHg	≤15 mmHg	
**BHS standard**	-	60.00%	85.00%	95.00%	A
	-	50.00%	75.00%	90.00%	B
	-	40.00%	65%	85%	C
[17]	SBP	59.46%	79.97%	88.85%	B
	DBP	76.95%	95.72%	99.97%	A
	MAP	-	-	-	-
[18]	SBP	50.07%	76.40%	90.39%	B
	DBP	65.66%	89.77%	96.63%	A
	MAP	65.14%	89.58%	96.61%	A
[19]	SBP	51.00%	81.00%	94.00%	B
	DBP	62.00%	92.00%	99.00%	A
	MAP	60.00%	90.00%	98.00%	A
[25]	SBP	67.66%	89.82%	96.82%	A
	DBP	82.79%	96.12%	99.09%	A
	MAP	84.21%	97.38%	99.58%	A
[23]	SBP	46.30%	72.10%	85.20%	C
	DBP	73.20%	91.90%	97.00%	A
	MAP	76.00%	92.30%	96.90%	A
[27]	SBP	76.21%	93.66%	97.71%	A
	DBP	93.51%	98.70%	99.46%	A
	MAP	-	-	-	-
This work	SBP	64.20%	87.85%	95.26%	A
	DBP	95.58%	99.35%	99.67%	A
	MAP	90.80%	98.61%	99.51%	A

**Table 5:** Comparison with related works based on real-time BP estimation.

Works	Method	Database	Input	RMSE	Pearson r	Real Time	Device Demonstration
[17]	BiLSTM	MIMIC-II	ECG, PPG (Features)	SBP: 8.051 DBP: 3.998 MAP: N/A	-	Yes	Yes
[18]	ResLSTM	MIMIC-III	ECG (Features)	-	SBP: 0.88 DBP: 0.71 MAP: 0.85	Yes	No
[19]	Multi-instance regression algorithm	Self-made	ECG, PPG (Features)	-	SBP: 0.90 DBP: 0.84 MAP: 0.88	Yes	Yes
[25]	CNN–LSTM	MIMIC-III	ECG, PPG (Raw)	-	SBP: 0.80 DBP: 0.85 MAP: 0.86	No	No
[23]	RDAE	MIMIC-II	PPG	-	-	No	No
[27]	U-net	MIMIC-I and MIMIC-III	PPG (Raw)	SBP: 5.75 DBP: 3.52 MAP: 3.75	SBP: 0.97 DBP: 0.96 MAP: N/A	No	No
This work	1D Squeeze U-net	MIMIC-I and MIMIC-III	PPG (Raw)	SBP: 6.50 DBP: 3.73 MAP: 4.10	SBP: 0.97 DBP: 0.96 MAP: 97	Yes	Yes

**Table 6:** Quantitative comparison between U-net and Squeeze U-net regarding model size, number of operations, and prediction time.

Model	#Parameters	Size (MB)	#Float Operations	Time/Prediction (ms)
U-net [27]	10,812,682	126.91	162,176,642	2.00
Squeeze U-net	819,921	101.44	1,637,678	0.32
Reduction Factor	13×	1.3×	99×	6.1×

## Data Availability

All the data used in this study are obtained from public datasets. Readers should be able to obtain those data by requesting the dataset sources described in this study.
